# Effect of TMR Briquettes on Milk Production, Nutrient Digestibility, and Manure Excretions of Dairy Cows in the Dry Zone of Sri Lanka

**DOI:** 10.3390/ani12070932

**Published:** 2022-04-05

**Authors:** Wishma Karunanayaka, Deepthi Nayananjalie, Ranga Appuhamy, Jayantha Adikari, Viraj Weerasingha, Amali Kumari, Sharini Somasiri, Ridma Liyanage, Priyani Mangalika, Thenmoli Sundarabarathy

**Affiliations:** 1 Department of Animal and Food Sciences, Rajarata University of Sri Lanka, Puliyankulama, Anuradhapura 50000, Sri Lanka; wishmakarunanayaka@gmail.com (W.K.); adikariamjb@agri.rjt.ac.lk (J.A.); vviraj@agri.rjt.ac.lk (V.W.); amalipubudu@agri.rjt.ac.lk (A.K.); sharinisc@agri.rjt.ac.lk (S.S.); thathsaraniridma@gmail.com (R.L.); 2 Department of Animal Science, Iowa State University, Ames, IA 50011, USA; appuhamy@iastate.edu; 3 Veterinary Research Institute, Gannoruwa, Peradeniya 20400, Sri Lanka; mangalikajasinghe@yahoo.com; 4 Department of Biological Sciences, Rajarata University of Sri Lanka, Mihintale 50300, Sri Lanka; tvbarathy@gmail.com

**Keywords:** blood serum, digestibility, lactating dairy cows, milk yield, TMR briquettes

## Abstract

**Simple Summary:**

Compact total mixed ration (CTMR) is a feeding concept to promote nutrition and productivity in cattle. We compared the milk production and nutrient digestibility of cows fed a form of CTMR, TMR briquettes made of dried forages vs. a conventional diet including fresh-cut Guinea grass in the dry zone of Sri Lanka. Our findings showed that feeding TMR briquettes tended to increase dry matter intake (DMI) and milk yield by about 0.5 and 1.0 kg/d, respectively. The TMR briquettes were related to higher fibre digestibility and lower milk urea nitrogen (MUN) and urinary nitrogen excretions compared to the conventional diet.

**Abstract:**

We showed previously that TMR briquettes made with a variety of forages and industrial by-products had higher crude protein and energy concentrations than the conventional diet including fresh-cut Guinea grass and commercial cattle pellet (CTL). The study objective was to determine to what extent the nutritional advantages of TMR briquettes would be translated into the milk production of dairy cows in the dry zone of Sri Lanka. Nine Jersey × Sahiwal cows were assigned to CTL or two TMR briquettes in a 3 × 3 Latin square design with three periods each including 14 d for production measurement and 7 d for total faeces and urine collection. The TMR briquettes tended to increase milk yield (5.55 to 6.59 kg/d, *p* = 0.092), milk protein yield (0.170 vs. 0.203 kg/d, *p* = 0.091) and DMI (6.50 to 7.16 kg/d, *p* = 0.070), and decreased milk urea nitrogen (13.0 to 10.5 mg/dL, *p* < 0.006). The TMR briquettes had a higher organic matter and neutral detergent fibre digestibility (*p* < 0.001), and lower urinary N excretions as a % of N intake (*p* = 0.149). In conclusion, the TMR briquettes can improve forage digestibility, milk production and environmental sustainability of dairy cows in the dry zone of Sri Lanka.

## 1. Introduction

Total mixed rations (TMR) offer a promising way to feed nutritionally balanced diets to dairy cows [1,2]. Total mixed rations also decrease feed wastage, and optimize the ruminal environment [3,4]. North American and European dairy producers widely use TMR formulated by using preserved forages. However, feeding TMR is not common in Sri Lanka, and the typical dairy cow diet mostly includes fresh-cut fodder, the availability of which depends on rainfall patterns. Based on rainfall and the availability of moisture conditions, Sri Lanka can be divided into three climatic regions: wet zone, intermediate zone, and dry zone. The dry zone is an important agro-climatic zone [5] housing the majority of the country’s dairy cow population [6]. The dry zone experiences a distinct pattern of dry and rainy seasons, and has a higher probability of encountering droughts than the wet zone [7]. As one would imagine, dry seasons are characterized by decreased forage yield and quality [6]. Rainy seasons, on the other hand, ensure surplus forage production that can be preserved for feeding cows in dry seasons. Preserved forages also alleviate the hardship of feeding cows in heavy rains. Moreover, most dairy producers hold day jobs [8], making it difficult to balance diets to meet nutrient requirements. Overall, a pre-balanced TMR made of preserved forage would be beneficial for dairy producers in the dry zone [9,10].

Compact TMR (CTMR) is a relatively new feeding concept [11] aiming to promote the nutrition, productivity and efficiency of cattle. The literature on CTMR highlights that there is less sorting, less frequent antagonistic feeding behaviour, and 1 to 2 kg/d higher milk yield compared to conventional TMR [11,12]. Moreover, compacting TMR into block-like structures known as TMR briquettes offers several advantages, including longer shelf life, lower storage space requirement, more efficient feed delivery, and decreased feed leftovers vs. conventional TMR [4,10]. The ability of CTMR to mitigate sorting is pertinent to the dry zone, as heat stress can exacerbate the sorting behaviours of cows [13]. Somasiri et al. [9] developed a TMR briquette using Gliricidia (*Gliricidia sepium*) forage and agro-industrial by-products such as coconut oil meal (also known as coconut poonac) and rice bran available in Sri Lanka. They observed increased dry matter intake (DMI) and milk yield (kg/cow/d) in cows fed Gliricidia briquettes compared to a conventional diet including grass and rice straw. Guinea grass (*Panicum maximum*) is the most abundant fodder species in the dry zone. Feeding fresh-cut Guinea grass (*Panicum maximum*) supplemented with commercial cattle pellets is common in the dry zone. Some producers also feed other forages such as maize (*Zea mays*), sorghum (*Sorghum bicolour*)*,* Napier (*Pennisetum purpureum* × *P. americanum*), and Gliricidia, and agro-industrial by-products such as rice bran, coconut poonac, and soybean meal [14]. We previously evaluated six TMR briquettes made with those ingredients with respect to their nutritional, physical, and microbiological properties during storage, which were similar among all TMR briquettes [10]. The cost of production for two briquettes was 15% less than that for the other briquettes [10]. The cost of production of the conventional diet (Guinea grass and commercial cattle pellets) providing the same amount of dry matter was nearly two times greater than that of the TMR briquettes. Moreover, TMR briquettes had 22 to 24% higher concentrations of crude protein (CP) and net energy of lactation (NEL) than the conventional diet [14]. We were keen on knowing to what extent those advantages of TMR briquettes would be translated into the milk production of local cows. The objective of this study was to determine the effects of TMR briquettes on DMI, milk production, and nutrient digestibility of dairy cows in comparison to the conventional diet. Additionally, we examined the dietary effects on faecal and urinary nitrogen excretions, data of which is limited for cattle in Sri Lanka.

## 2. Materials and Methods

### 2.1. Experimental Site

This experiment was conducted at the livestock experiment farm of Rajarata University of Sri Lanka (Puliyankulama, Sri Lanka) under the approval (VERC-19-09) of the Animal Ethics Committee of Faculty of Veterinary Medicine and Animal Science at University of Peradeniya (Peradeniya, Sri Lanka). The analyses of feeds, faeces, milk, blood and urine described below were conducted in the Department of Animal and Food Sciences at Rajarata University of Sri Lanka (Puliyankulama, Sri Lanka).

### 2.2. Animals and Treatments

Nine Jersey × Sahiwal crossbred dairy cows with 275 ± 33 kg (mean ± standard deviation) body weight (BW) and 4.6 ± 0.7 kg/d milk production at 14 d in milk were purchased from local farmers. After being adapted to the experimental location for 14 d, cows were assigned randomly to one of three treatments in a replicated 3 × 3 Latin square design (LSD) consisting of three 35 d experimental periods separated by 14 washouts. Each experimental period consisted of 14 d for treatment adaptation followed by 14 d production measurement and then 7 d faeces and urine collection measurements. The three treatments were the control diet including fresh-cut and chopped (2.5 cm) Guinea grass (*Panicum maximum*) mixed with a commercially prepared cattle feed pellet (CTL), and two TMR briquettes that were prepared using a variety of locally available forages (dried and chopped) and industrial by-products (TMR1 and TMR2). The CTL was fed to all cows during the washout. The ingredient and nutrient composition of treatment diets are presented in Table 1 and Table 2, respectively.

Our previous publications [10,14] comprehensively describe the TMR briquettes. Briefly, six TMR briquettes were prepared by using different combinations of locally available forages and industrial by-products to meet 110% of the nutritional requirements of local dairy cows (DMI = 9.0 kg/d, BW = 300 kg, milk yield = 10 kg/d, and milk fat = 4.5%) according to the NRC [15]. Forages were chopped into 2.5 cm pieces by using a grass chopper (CHC 93 ZT, Henan Wisely, Kaifeng, China) and air-dried to decrease moisture content to 20%. Agro-industrial by-products were ground into a powder. The ingredients in each ration were mixed for 5 min at 26 rpm by using a feed mixture (Vimamix, Vietnam Agro Tech, Ho Chi Minh, Vietnam). Each TMR mixture was compacted into briquettes of 10 kg (as-fed weight) using a hydraulic briquette press (Green Pack 09, Christo, Katana, Sri Lanka) and the briquettes were wrapped with polythene (gauge 300). The average cost of production of TMR briquettes including ingredient, labour, and electricity costs was LKR 34.20 per 1.0 kg of DM. This cost represents about 50% of the price producers receive for 1.0 L of milk. The production cost of TMR1 and TMR2 were 15% less than that of the other TMR briquettes and 50% less than the cost of CTL [10,14].

Cows were housed individually in metabolic stalls (1.1 m × 1.6 m) throughout the study (December 2020 to April 2021). The TMR briquettes and CTL were offered daily in two equal meals at 08:00 and 16:00 h. Each cow had ad libitum access to drinking water. The barn had four fans, and each stall was equipped with a water sprinkler that was turned on when the environmental temperatures increased above 30 °C. A field veterinarian inspected cows once per week throughout the study.

### 2.3. Total Collection of Faeces and Urine

The total faeces and urine outputs were collected daily in the last week of each period. Cows were fitted with urinary catheters (20 mm Foley Catheter, Zhanjiang Star Enterprise Co., Ltd., Zhanjiang, China) and allowed to adapt to it during the next 24 h. The catheter end was connected to a tube running to a collection jar. Urine volume was recorded, and a 50 mL urine sample was collected at 10:00 and 16:30 h. The urine samples were acidified with 1.0 mL of 6.0 N H_2_SO_4_ and stored at −18 °C until being analysed for urinary nitrogen (N). Cows lost catheters in four incidents, three of which could be corrected within 30 min. Correction of the other incident took several hours. Therefore, the data of that cow on that day was not included in the statistical analysis. Faeces was scraped into plastic crates, and faeces weight was recorded daily at 10:00 and 16:30 h. A sample representative of faeces collected during the whole day was stored in plastic bags at −18 °C. At the end of each period, those faeces samples were thawed and mixed to obtain a composited sample (~250 g) for each cow within each period. The composited samples were freeze-dried (Alpha 2-4 Lse basic, Martin Christ GmbH, Osterode am Harz, Germany), ground, and stored in sealed plastic bottles at room temperature until being analysed for crude protein (CP), neutral detergent fibre (NDF), acid detergent fibre (ADF) and ash contents.

### 2.4. Animal Measurements, Sample Collection, and Analyses

The BW was measured by using an electronic cattle weighing bridge (AS-1-ASS, Qingdao, China) immediately after the morning milking in the beginning of the study and once in every week afterward. Body condition score (BCS) was assessed by a field veterinarian using a scale of 1 to 5 [16] immediately after every body weight measurement. A device similar to the Penn State Particle Separator (PSPS) was used to determine the particle size distribution of treatment diets. The top, middle, and lower tray (30 cm × 30 cm) sieve sizes were 19.0, 8.0, and 3.0 mm, respectively. A 1.0 kg sample of each diet was shaken and the particles remaining in trays and the bottom pan were weighed separately. Physically effective NDF (peNDF) content was calculated using those records and the NDF content (% of DM) according to Damery et al. [17].

The weight of feed offered to and refused by individual cows was recorded daily. A sample of offered diet was saved weekly at −20 °C, and composited for individual cows within each period at the end of the study. The composite samples were oven-dried (YCO-010, Gemmi Industrial Corporation, Taipei, Taiwan) at 60 °C for 48 h, ground (1.0 mm), and stored in plastic bottles at room temperature. Nutrient composition of feed and faeces samples was analysed by using the AOAC [18] procedures; ash by using a muffle furnace (DMF-05, HumanLab Instrument Co., Gyeonggi-do, Korea), and CP by using a Kjeldahl digestion unit (DK 20, Velp Scientific, Usmate Velate, Italy). Acid detergent fibre and NDF were analysed by using fibre analyser (FIWE3, Velp Scientific, Usmate Velate, Italy) according to Van Soest et al. [19].

Cows were milked twice a day at 06:00 and 15:00 h using a portable milking machine (InterPuls, Italy). Milk volume was recorded at each milking to calculate the daily milk yield per cow. Milk samples (50 mL) were collected from each cow once every three days and stored at −18 °C. At the end of each period, milk samples were thawed and mixed to obtain a 50 mL composite sample for each cow within each period. The composite samples were analysed for fat, true protein, and milk urea nitrogen (MUN) concentrations. Fat and true protein concentrations were analysed using an automated milk analyser (Lactoscan SP, Nova Zagora, Bulgaria). The MUN concentration was determined using a modified colorimetric p-dimethyl amino benzaldehyde (DMAB) assay kit (Sigma Aldrich, Buchs, Switzerland) and a UV-visible spectrophotometer (Orion aquamate 8000, VWR International, LLC, Radnor, PA, USA) at 425 nm wavelength. Blood samples (10 mL) were collected from the jugular vein into vacuum tubes containing K3-EDTA at the beginning and the end of each period. Blood samples were centrifuged at 3000× *g* for 10 min (Model-C0060, Edison, NJ, USA), and serum was transferred into the Eppendorf tubes stored at −18 °C. The serum was analysed in triplicate for glucose (GLUC-PAP ELISA kit, Randox Laboratories, Crumlin, UK), blood urea nitrogen (BUN; UREA ELISA kit, Randox Laboratories, Crumlin, UK), β-hydroxybutyrate (BHBA; RANBUT ELISA kit, Randox Laboratories, Crumlin, UK), non-esterified fatty acids (NEFA; E-BC-K014 ELISA kit, Elabscience, Wuhan, China), albumin (E-EL-R0362 ELISA kit, Elabscience, Wuhan, China) using commercial kits and a microplate spectrophotometer (51119700DP, Thermo Scientific, Waltham, MA, USA) in accordance with the manufacturer’s protocols. The intra-assay coefficients of variance were <14.0% for all the assays.

### 2.5. Calculations and Statistical Analysis

Nutrient intake (*N_i_*, kg/cow/d) was calculated by multiplying DMI by corresponding nutrient content (Table 2). Faecal DM output was calculated by multiplying fresh faeces output by faecal DM content. The faecal outputs of individual nutrients (*N_f_*, kg/cow/d) were calculated by multiplying faecal DM output by corresponding nutrient content in faeces. Apparent total tract digestion (*ATTD*) of ith nutrient was calculated as follows.

$$ATTD_{i}=\frac{N_{i}-N_{f}}{N_{i}}$$

Energy corrected milk (ECM, kg/d) was calculated using the following equation [20].

ECM =(0.327×milk yield, kg/d)+(12.95×milk fat, kg/d)+(7.65×milk protein, kg/d)

Milk protein efficiency (MPE) was calculated by expressing the milk protein yield (kg/d) as a percentage of CP intake (kg/d).

Treatment effects on DMI, milk yield and composition, dietary nutrient composition, BW, BCS, dietary particle distribution, serum metabolite concentrations, *ATTD*, and manure N excretions were determined using the MIXED procedure of SAS (version 9.0, SAS Institute Inc., Cary, NC, USA) according to the following model:

Y_ijkl_ = µ + T_i_ + P_j_ + S_k_ + C_l_ (S_k_) + e_ijkl_

where Y_ijkl_ = the response variable of interest, μ = overall mean, T_i_ = fixed effect of ith treatment (i = CTL, TMR1, and TMR2), P_j =_ fixed effect of jth period (j = 1 to 3), S_k_ = random effect of kth treatment sequence (k = 1 to 3), C_l_ (S_k_) = random effect of lth cow nested in kth treatment sequence, and e_ijkl_ = random error assumed to be independent and identically distributed. Plasma metabolite concentrations at the beginning of each period were included in the model as a covariate when treatment effects on plasma metabolites were analysed. The least squares means were compared using the Tukey test. Statistically significant differences, tendencies, and numerical differences were declared at *p* ≤ 0.05, 0.05 < *p* ≤ 0.10, and 0.10 < *p* ≤ 0.20, respectively.

## 3. Results and Discussion

The seasonality of forage and industrial by-product availability are constraints in achieving the milk production potential of dairy cows (e.g., Jersey × Sahiwal) in the dry zone of Sri Lanka [6]. The lack of producer awareness of ration balancing is another constraint [21]. The TMR briquettes promote forage preservation (e.g., drying) and the utilization of preserved forages in formulating nutritionally balanced diets that are compacted to store with minimum space requirements for several months [22]. Our previous examinations confirmed that CP, NDF, and ADF concentrations of locally produced TMR briquettes remained unchanged over four-month storage [10]. We present and discuss below production performance, important blood metabolites, nutrient digestibility [23], and manure nitrogen excretions of cows fed those TMR briquettes vs. a conventional diet in the dry zone of Sri Lanka.

### 3.1. Particle Size Distribution of Diets

The particle size distribution of treatment diets is presented in Table 3. The percentages of feed particles >8.0 mm was higher (*p* < 0.001) in CTL compared to TMR1 and TMR2. The percentage of feed particles between 8.0 and 3.0 mm was higher in TMR2 compared to that of TMR1 (*p* = 0.037). The TMR briquettes had nearly 10 times greater percentages of the smallest particles than CTL (<3.0 mm, *p* < 0.001). Again, TMR1 had a higher percentage of <3.0 mm particles than TMR2 (*p* = 0.020). Large forage particles enhance rumen fill, chewing time, and saliva production [24,25]. On the other hand, dry diets with large forage particles can encourage the sorting behaviour of cattle [26,27]. Nevertheless, we could not assess sorting behaviour, because the particle distribution of feed refusals had not been recorded. Owing to higher NDF content and large particle percentages, CTL had a higher peNDF than TMR1 and TMR2 (*p* < 0.010). The peNDF measures the diet’s ability to stimulate chewing activity and thus ruminal buffering by saliva [28]. In theory, peNDF is calculated by using the percentage of feed retained above the 4.0 mm sieve [17]. Using a lower sieve size (3.0 mm) could be a reason the present peNDF values were higher than the values (27.5 to 30.4%) of Oh et al. [29]. Nevertheless, the peNDF across treatments were about two times higher than the range recommended for lactating dairy cows [28]. Unnecessarily high peNDF could decrease feed efficiency [30]. Nevertheless, the relationships among tropical forages, peNDF, and feed efficiency are yet to be fully explored.

### 3.2. Dry Matter Intake, Milk Production and Body Measurements

Table 4 presents DMI, milk yield, and milk component yields. Cows consuming TMR briquettes tended to have a greater DMI than CTL (*p* = 0.070). The differences in the DM content of the diets (Table 2) postulate a fresh feed intake three times greater for CTL than for TMR briquettes. Therefore, the decreased DMI of CTL could be a result of increased rumen fill compared to TMR briquettes [31,32]. Aligning with the DMI increment, milk yield (kg/d) tended to increase in response to TMR2 by about 1.0 kg/d compared to CTL. In support, Sarkar et al. [2] demonstrated that cows fed TMR briquettes produced more milk than cows fed fresh forages supplemented with concentrates. The average milk production of our cows falls within the range (6 to 10 kg/d) of Sahiwal × Jersey cows [33], but is lower than purebred Jersey cows in Sri Lanka [34]. Milk protein yield tended to increase by 15% (*p* = 0.091) for TMR briquettes vs. CTL, possibly because of the increased milk yield, as milk protein concentrations were similar among treatments (*p* = 0.244). Milk urea nitrogen decreased for TMR2 compared to CTL (*p* < 0.010) suggesting improved milk protein efficiency, whole-body protein efficiency, or both [35]. The TMR briquettes seemed to improve the energy balance of cows as indicated by numerically higher BCS compared to CTL (*p* = 0.198). Nevertheless, ECM were not different among treatments suggesting similar efficiencies of utilizing energy for milk production.

### 3.3. Plasma Metabolites

Plasma concentrations of glucose, BUN, NEFA, ALB, and BHBA are presented in Table 5. None of those parameters were affected by dietary treatments. Nevertheless, the average glucose concentration of our cows was lower than that of Jersey × Sahiwal cows reported in Sreedhar et al. [36]. This discrepancy could be due to differences in multiple factors such as environmental temperature and the stage of lactation between studies [37]. The BUN values are within the range of Jersey × Sahiwal cows in the tropical environment (20.5 and 25.4 mg/dL; [36]). BUN less than 15.0 mg/dL is typical for European cows exhibiting high nitrogen utilization efficiency [38]. Therefore, the present BUN at 20.0 mg/dL could reflect poor nitrogen utilization efficiency across treatments [38,39]. Elevated NEFA and BHBA indicate fatty acid mobilization from adipose tissues in response to a negative energy balance, common among high-producing dairy cows [40,41]. The present NEFA concentrations (5.53 to 8.78 mg/dL) were well below even the concentrations of high-producing cows with desired energy balance (14.0 mg/dL; [42]). Although the relationships between energy balance and the blood metabolites of Jersey × Sahiwal cows have yet to be fully understood, given the production level, it was unlikely that our cows experienced a negative energy balance. The groups fed TMR briquettes tended to have a higher plasma albumin concentration than CTL (*p* = 0.062). High plasma albumin concentrations could indicate increased plasma protein synthesis by the liver. Perhaps TMR briquettes with higher CP concentrations provided more precursors to synthesize liver proteins than the CTL. Moreover, the baseline blood metabolite concentrations measured on the first day of the measurement period were not different between periods suggesting the 14 d washout period was adequate.

### 3.4. Nutrient Intake and Digestibility

The nutrient intake (kg/d) and apparent total tract digestibility of nutrients are presented in Table 6. The DMI was similar at 6.95 kg/d between treatments during the digestibility trial. Therefore, the differences in nutrient contents (Table 2) dictated the differences in nutrient intake between diets (*p* < 0.001, Schroeder et al. [43]). In line with higher CP and lower ash contents, TMR1 and TMR2 had greater CP intake and lower ash intake than CTL. Moreover, ADF intake differences were a mirror image of the differences in dietary ADF concentrations. Similar to DMI, NDF and organic matter intake were not significantly different between treatments. Kendall et al. [44] reported a stronger correlation of NDF intake with DMI than the NDF concentration. The DM digestibility of both TMR briquettes was higher compared to CTL (0.573 and 0.626 vs. 0.440, *p* < 0.001, Table 6). Consistently, Sarker et al. [2] observed higher DM digestibility (0.646 vs. 0.535) for compact TMR made of maize stover and industrial by-products (wheat bran, lentil bran, soybean meal, and molasses) compared to Napier grass supplemented with the same concentrates in tropical cows. On the other hand, Petters [24] reported no change in DM digestibility for compact TMR vs. traditional TMR in Swedish Holstein cows. The impact of compact TMR on DM digestibility seems to vary from region to region depending on the diet composition and breed of cows. Man and Wiktorsson [45] and Raharjo [46] reported similar DM, NDF, and ADF digestibility for grass species used in the present study. However, Serasinhe and Pathirana [47] showed that the DM digestibility of a mixture of forages was greater than that of the individual forages. Therefore, treatment effects on nutrient digestibility could be confounded in forage composition in the present study.

The digestibility of ash in TMR2 was higher than CTL (*p* = 0.029). The digestibility of organic matter in both TMR1 and TMR2 was higher than CTL (*p* < 0.001). The improved organic matter digestibility of TMR briquettes could be linked with high CP contents (Table 2) shown to enhance the organic matter digestibility of dairy cows [48,49]. The digestibility of CP was, however, similar at 68.7% across the diets (*p* = 0.232). Drying could offset potential improvements of CP digestibility. Puchala [50] observed a decreased CP digestibility for feeding hay vs. feeding fresh forage. The ADF and NDF digestibility of TMR briquettes were higher than that of CTL (*p* < 0.001). Drying forages was able to increase the NDF digestibility, which in turn contributes to organic matter digestibility [51,52]. Overall, TMR briquettes seem to provide more digestible energy than CTL by improving fibre digestibility.

### 3.5. Manure Excretions and N Efficiencies

Table 7 presents manure volumes and N excretions. The TMR2 had a lower faeces output (kg/d) than CTL (*p* = 0.028). Both TMR diets were associated with greater faecal N excretions (g/d, *p* < 0.010) potentially due to higher CP intake than CTL. Nevertheless, the % of N intake excreted in faeces was similar between treatments as also indicated by the CP digestibility similar between treatments (*p* = 0.232). Urine volume (L/d) increased for TMR1 compared to TMR2 even though both had similar N intake and N digestibility shown to regulate urine output of dairy cows [53]. Differences in other factors affecting urine output such as dietary electrolytes concentrations and drinking water intake could be responsible for the urine volume difference between TMR1 and TMR2 [53]. Cows consuming TMR2 were related to numerically lower % of dietary N excreted in urine than CTL (25.07 vs. 41.90%, *p* = 0.149) suggesting improved N utilization efficiency, which was also reflected in MUN. Nevertheless, MPE was similar at 24% (*p* = 0.221) among treatments, suggesting that milk protein synthesis wouldn’t be a part of the improved N utilization efficiency. Although the TMR briquettes likely provide more metabolizable protein due to higher CP concentrations than CTL, potential limitations of individual essential amino acid supply for milk protein synthesis could hamper MPE [54].

## 4. Conclusions

For the first time, TMR briquettes made with multiple forages (dried and chopped) were evaluated against a conventional diet (CTL) made with Guinea grass (fresh and chopped) for milk production, N efficiency, and manure N excretions of dairy cows in the dry zone of Sri Lanka. TMR briquettes exhibited increased NDF and ADF digestibility compared to the CTL. The TMR briquettes increased milk yield and milk protein yield by 14 to 15% compared to the CTL. The MUN and urinary N (% of N intake) decreased in response to feeding TMR briquettes compared to CTL. The TMR briquettes and CTL had similar milk protein efficiency. Overall, TMR briquettes seem to offer a promising way to preserve forages with improved fibre digestibility that could increase the milk and milk protein yield of cows in the dry zone of Sri Lanka. Balancing the TMR for amino acid and micronutrient requirements could improve production performance and efficiency.

## Figures and Tables

**Table 1:** Ingredient composition (g/kg of DM) of treatment diets ^1^.

Ingredients	CTL	TMR1	TMR2
Forages			
Gliricidia (*Gliricidia sepium*)		085	110
Guinea *grass* (*Panicum maximum*)	650	215	140
Maize (*Zea mays*)		160	110
Napier grass ^2^		130	190
Sorghum (*Sorghum bicolor*)		130	215
Concentrates			
Rice (*Oryza sativa)* bran		100	065
Ground maize (*Zea mays*)		025	070
Soybean (*Glycine max)* meal		025	030
Coconut (*Cocos nucifera)* poonac		110	050
Mineral mixture		020	000
Dicalcium phosphate			020
Commercial cattle feed ^3^	350		

^1^ CTL = conventional diet; TMR1 and TMR2 = total mixed ration briquettes made of dried and chopped forages. ^2^ *Pennisetum purpureum × Pennisetum americanum*. ^3^ Cereals, cereal by-products, oil seed meal, vegetable oil, and a mineral and vitamin mix.

**Table 2:** Nutrient composition (mean ± SD) of control diet and total mixed ration (TMR) briquettes.

Variable ^2^	Treatments ^1^		
	CTL	TMR1	TMR2
DM, g/kg	256 ± 21.2	889 ± 14.0	886 ± 10.7
Ash, g/kg	133 ± 5.9	111 ± 16.8	111 ± 26.8
CP, g/kg	94.3 ± 8.8	116 ± 10.6	115 ± 10.0
ADF, g/kg	438 ± 32.1	345 ± 23.4	387 ± 25.8
NDF, g/kg	527 ± 37.0	488 ± 33.0	486 ± 26.6
NEL, Mcal/kg of DM	0.960 ± 0.047	1.21 ± 0.040	1.18 ± 0.031

^1^ CTL = conventional diet including a mixture of fresh-cut grasses and a commercial cattle pellet; TMR1 and TMR2 = total mixed ration briquettes made of dried and chopped forages, and locally available industrial by-products. ^2^ DM = dry matter, CP = crude protein, ADF = acid detergent fibre, NDF = neutral detergent fibre, and NEL = net energy of lactation.

**Table 3:** Particle size distribution (%) of the diets offered to cows.

Variable ^2^	Treatments ^1^			SEM	*p*-Value
	CTL	TMR1	TMR2		
Particle size distribution					
>19 mm	71.6 ^a^	48.8 ^b^	49.7 ^b^	1.01	<0.001
19 mm–8 mm	24.2 ^a^	21.6 ^b^	21.3 ^b^	0.55	0.009
8 mm–3 mm	1.65 ^c^	5.18 ^b^	6.62 ^a^	0.414	<0.001
<3 mm	2.55 ^c^	24.4 ^a^	22.4 ^b^	0.485	<0.001
peNDF	51.4 ^a^	36.9 ^b^	37.7 ^b^	0.36	<0.001

^1^ CTL = cows eating the conventional diet including a mixture of fresh-cut grasses and a commercial cattle pellet; TMR1 and TMR2 = cows eating total mixed ration briquettes made of dried and chopped forages, and locally available industrial by-products. ^2^ peNDF = Physically effective neutral detergent fibre. ^a–c^ Different letters in the same row denote different treatment means (*p* < 0.05).

**Table 4:** Dry matter intake, milk production performance and body measurements.

Variable ^2^	Treatments ^1^			SEM	*p*-Value
	CTL	TMR1	TMR2		
DMI, kg/d	6.50	7.16	7.00	0.202	0.070
Milk yield, kg/d	5.55	6.59	6.04	0.558	0.092
Fat, g/kg	38.4	34.5	37.6	2.05	0.354
Fat, kg/d	0.218	0.221	0.225	0.0253	0.961
Protein, g/kg	30.6	30.7	31.4	0.34	0.244
Protein, kg/d	0.170	0.203	0.188	0.0171	0.091
MUN, mg/dL	13.0 ^a^	13.0 ^a^	10.5 ^b^	0.65	0.006
ECM, kg/d	5.94	6.57	6.32	0.623	0.519
BCS	2.47	2.63	2.60	0.084	0.198
BW, kg	259	271	270	9.6	0.326

^1^ CTL = cows eating the conventional diet including a mixture of fresh-cut grasses and a commercial cattle pellet; TMR1 and TMR2 = cows eating total mixed ration briquettes made of dried and chopped forages, and locally available industrial by-products. ^2^ DMI = dry matter intake, MUN = milk urea nitrogen, ECM = energy corrected milk, BCS = body condition score, BW = body weight. ^a,b^ Different letters in the same row denote different treatment means (*p* < 0.05).

**Table 5:** Concentrations of blood plasma parameters.

Variable ^2^	Treatments ^1^			SEM	*p*-Value
	CTL	TMR1	TMR2		
Glucose, mg/dL	63.3	61.9	64.2	1.59	0.630
BUN, mg/dL	20.7	20.4	19.6	0.97	0.726
NEFA, mg/dL	8.14	5.53	8.78	1.311	0.210
ALB, ng/mL	1.69	1.95	2.10	0.110	0.062
BHBA, mg/dL	3.80	3.51	2.41	0.415	0.664

^1^ CTL = cows eating fresh forages and a cattle feed, and TMR1 and TMR2 = cows eating total mixed ration briquettes made of dried and chopped forages, and locally available industrial by-products; ^2^ BUN = blood urea nitrogen, NEFA = non-esterified fatty acids, ALB = albumin, BHBA = ß-hydroxybutryrate.

**Table 6:** Intake and apparent total tract digestibility of nutrients.

Variable ^2^	Treatments ^1^			SEM	*p*-Value
	CTL	TMR1	TMR2		
Nutrient intake, kg/d					
DM	6.8	7.06	6.99	0.213	0.57
Ash	0.917 ^a^	0.781 ^b^	0.774 ^b^	0.0251	<0.001
OM	5.88	6.28	6.22	0.187	0.194
CP	0.639 ^b^	0.819 ^a^	0.801 ^a^	0.0242	<0.001
ADF	2.98 ^a^	2.44 ^c^	2.71 ^b^	0.081	<0.001
NDF	3.58	3.45	3.4	0.105	0.331
Nutrient digestibility					
DM	0.440 ^b^	0.573 ^a^	0.626 ^a^	0.0189	<0.001
Ash	0.370 ^b^	0.414 ^ab^	0.493 ^a^	0.0282	0.029
OM	0.450 ^b^	0.592 ^a^	0.641 ^a^	0.0182	<0.001
CP	0.703	0.665	0.692	0.0159	0.232
ADF	0.416 ^c^	0.491 ^b^	0.617 ^a^	0.0219	<0.001
NDF	0.418 ^c^	0.582 ^b^	0.656 ^a^	0.0183	<0.001

^1^ CTL = cows eating the conventional diet including a mixture of fresh-cut grasses and a commercial cattle pellet; TMR1 and TMR2 = cows eating total mixed ration briquettes made of dried and chopped forages, and locally available industrial by-products. ^2^ DM = dry matter, OM = organic matter, CP = crude protein, ADF = acid detergent fibre, and NDF = neutral detergent fibre. ^a–c^ Different letters in the same row denote different treatment means (*p* < 0.05).

**Table 7:** Faecal and urinary nitrogen (N) outputs.

Variable	Treatments ^1^			SEM	*p*-Value
	CTL	TMR1	TMR2		
Fresh faeces volume, kg/d	17.9 ^a^	15.6 ^ab^	14.3 ^b^	0.83	0.028
Faecal N, g/d	30.5 ^b^	44.2 ^a^	39.6 ^a^	2.44	0.004
Faecal N, % of N intake	29.7	33.5	30.8	1.60	0.232
Urine volume, L/d	8.60 ^b^	10.2 ^a^	8.98 ^b^	0.578	0.008
Urinary N, g/d	43.1	37.0	31.8	7.34	0.568
Urinary N, % of N intake	41.9	27.2	25.1	6.21	0.149
Faecal N + Urinary N, g/d	73.6	81.2	71.5	7.66	0.652
Faecal N + Urinary N, % of N intake	71.6	60.7	55.8	6.43	0.247
Milk protein efficiency, %	25.9	24.4	22.9	2.13	0.221

^1^ CTL = cows eating the conventional diet including a mixture of fresh-cut grasses and a commercial cattle pellet; TMR1 and TMR2 = cows eating total mixed ration briquettes made of dried and chopped forages, and locally available industrial by-products. ^a,b^ Different letters in the same row denote different treatment means (*p* < 0.05).

## Data Availability

All datasets collected and analysed during the current study are available from the corresponding author on fair request.
